# Autoantigen-specific immunosuppression with tolerogenic peripheral blood cells prevents relapses in a mouse model of relapsing-remitting multiple sclerosis

**DOI:** 10.1186/s12967-016-0860-6

**Published:** 2016-05-01

**Authors:** Christian Kleist, Elisabeth Mohr, Sadanand Gaikwad, Laura Dittmar, Stefanie Kuerten, Michael Platten, Walter Mier, Michael Schmitt, Gerhard Opelz, Peter Terness

**Affiliations:** Department of Transplantation Immunology, Institute for Immunology, University of Heidelberg, Im Neuenheimer Feld 305, 69120 Heidelberg, Germany; Clinical Cooperation Unit Neuroimmunology and Brain Tumor Immunology, German Cancer Research Center (DKFZ), Im Neuenheimer Feld 280, 69120 Heidelberg, Germany; Department of Anatomy I, University of Cologne, Joseph-Stelzmann-Str. 9, 50931 Cologne, Germany; Department of Neurooncology, University of Heidelberg, Im Neuenheimer Feld 400, 69120 Heidelberg, Germany; Department of Radiology, Division of Nuclear Medicine, University of Heidelberg, Im Neuenheimer Feld 400, 69120 Heidelberg, Germany; Department of Internal Medicine V, University of Heidelberg, Im Neuenheimer Feld 410, 69120 Heidelberg, Germany; Department of Radiology, Division of Nuclear Medicine, University of Heidelberg, 69120 Heidelberg, Germany; Hexal AG, 83607 Holzkirchen, Germany; Quintiles GmbH, 63263 Neu-Isenburg, Germany; Becton Dickinson GmbH, BD Life Sciences, 69120 Heidelberg, Germany; Department of Anatomy and Cell Biology, University of Wuerzburg, 97070 Würzburg, Germany

**Keywords:** Autoimmunity, Cell therapy, Copaxone^®^, Immune tolerance, Mitomycin C, Relapsing-remitting MS, Regulatory T cells

## Abstract

**Background:**

Dendritic cells (DCs) rendered suppressive by treatment with mitomycin C and loaded with the autoantigen myelin basic protein demonstrated earlier their ability to prevent experimental autoimmune encephalomyelitis (EAE), the animal model for multiple sclerosis (MS). This provides an approach for prophylactic vaccination against autoimmune diseases. For clinical application such DCs are difficult to generate and autoantigens hold the risk of exacerbating the disease.

**Methods:**

We replaced DCs by peripheral mononuclear cells and myelin autoantigens by glatiramer acetate (Copaxone^®^), a drug approved for the treatment of MS. Spleen cells were loaded with Copaxone^®^, incubated with mitomycin C (MIC_Cop_) and injected into mice after the first bout of relapsing-remitting EAE. Immunosuppression mediated by MIC_Cop_ was investigated in vivo by daily assessment of clinical signs of paralysis and in in vitro restimulation assays of peripheral immune cells. Cytokine profiling was performed by enzyme-linked immunosorbent assay (ELISA). Migration of MIC_Cop_ cells after injection was examined by biodistribution analysis of ^111^Indium-labelled MIC_Cop_. The number and inhibitory activity of CD4^+^CD25^+^FoxP3^+^ regulatory T cells were analysed by histology, flow cytometry and in vitro mixed lymphocyte cultures. In order to assess the specificity of MIC_Cop_-induced suppression, treated EAE mice were challenged with the control protein ovalbumin. Humoral and cellular immune responses were then determined by ELISA and in vitro antigen restimulation assay.

**Results:**

MIC_Cop_ cells were able to inhibit the harmful autoreactive T-cell response and prevented mice from further relapses without affecting general immune responses. Administered MIC_Cop_ migrated to various organs leading to an increased infiltration of the spleen and the central nervous system with CD4^+^CD25^+^FoxP3^+^ cells displaying a suppressive cytokine profile and inhibiting T-cell responses.

**Conclusion:**

We describe a clinically applicable cell therapeutic approach for controlling relapses in autoimmune encephalomyelitis by specifically silencing the deleterious autoimmune response.

## Background

Multiple sclerosis (MS) is a chronic inflammatory demyelinating disease of the central nervous system. Although many aspects of the etiology and pathogenesis of disease have not been exhaustively clarified, there is no doubt that the immune system plays a major role in the brain-damaging process [1]. Several therapeutic tools offer the possibility of inhibiting the immune response, thus controlling the pathogenic process of multiple sclerosis. This however, happens at the expense of an undifferentiated immunosuppression leading to side effects [2].

Special attention merit novel promising approaches, such as treatment of MS patients with fumarates or laquinimod. Dimethylfumarate was effective in both MS and psoriasis [3, 4]. Recent studies by Ghoreschi et al. showed that improvement of diseases occurs by induction of type II dendritic cells (DCs) which produce IL-10 instead of IL-12 and IL-23 [5]. Laquinimod strongly reduced infiltration of CD4^+^ and CD8^+^ T cells in the central nervous system and prevented relapses of EAE in mice. Based on observations in mice and humans Jolivel et al. hypothesized that this beneficial effect was mediated by DCs [6]. A couple of therapeutic attempts envisage the suppression of the brain-damaging attack without affecting the remaining immune response [7]. These strategies include: administration of attenuated autoreactive T cells, T-cell receptor peptide vaccination, DNA-vaccination, treatment with altered peptide ligands, vaccination against axonal growth inhibitors associated with myelin or the use of DCs pulsed with specific antigens [7].

Our previous animal studies showed that incubation of DCs with the chemotherapeutic agent mitomycin C (MMC) can convert these strongly stimulatory cells into suppressive cells. In a rat heart transplant model, pretreatment of recipients with donor DCs incubated with MMC induced suppression of allograft rejection [8]. In an attempt to explore whether this approach is also applicable to prevention of autoimmune diseases, syngeneic DCs were loaded with myelin basic protein (MBP), an autoantigen derived from the brain, incubated with MMC and then injected into mice [9]. The animals became resistant to subsequently induced experimental autoimmune encephalomyelitis (EAE), demonstrating that autoantigen-loaded, MMC-treated DCs can be used as a protective vaccine in autoimmune diseases—a finding which is in line with other observations [10]. Here, we now address the question whether autoantigen-loaded suppressive cells might also be used for the therapy of an ongoing disease.

In a clinical setting the described cell therapeutic approach would raise some critical points. Injection of MBP or other autoantigens to a patient with MS entails the risk of exacerbating the disease. In the 1970s, a random copolymer of amino acids, termed “glatiramer acetate” (GA; Copaxone^®^, Cop), was initially developed to mimic the composition of MBP [11]. Studies revealed that this copolymer has various targets within the immune response, among others interfering with T-cell reactions against some myelin antigens, such as MBP or proteolipid protein (PLP) [12–14]. In clinical trials, Cop slowed the progression of disability and reduced the relapse rate of MS [15, 16] eventually resulting in its approval for the treatment of relapsing-remitting MS. Based on all these findings, the brain autoantigen—one hazardous component of our cell therapeutic—was replaced with Cop.

Moreover, naive DCs—the cells loaded with autoantigens—are strongly stimulatory cells, and even if rendered suppressive, they might regain their stimulatory capacity in vivo, thus leading to an activation of the disease. Apart from the risk of immunostimulation, generation of DCs is rather time-consuming, expensive and difficult to standardize. Therefore, we replaced DCs with syngeneic PBMCs—cells which are less immunogenic and much easier to prepare.

In the present work, the therapeutic properties of Cop-loaded MMC-induced peripheral blood mononuclear cells (MIC_Cop_) were analyzed in mice with EAE. The findings pave the way for targeted immunosuppression in patients with MS and other autoimmune diseases.

## Methods

### Animals and EAE model

SJL/J mice (females, 6–8 weeks of age; haplotype H-2^s^) were purchased from Charles River Laboratories (Sulzfeld, Germany) and Janvier Labs (Le Genest-Saint-Isle, France) and kept at the Interfacultary Biomedical Research Facility (IBF) of the University of Heidelberg (Heidelberg, Germany). SJL/J mice were used for an EAE model of relapsing-remitting disease after administration of immunogenic proteolipid protein (PLP) peptide PLP_139–151_. Mice received a standard rodent diet and water ad libitum. During EAE experiments, food pellets as well as wet food and water (also supplied as water gel pouches) were placed on the floor of the cages as soon as clinical paralytic signs had been observed. The project was approved by the Animal Welfare Board of the Governmental Office (Karlsruhe, Germany) and the University of Heidelberg Committee for Ethics on Laboratory Animal Experimentation and was performed in compliance with institutional guidelines, the German law for animal protection, the Directive 2010/63/EU of the European Union on the protection of animals used for scientific purposes and FELASA (Federation of European Laboratory Animal Science Associations, Ispwich, UK) guidelines and recommendations.

### Induction of EAE and clinical assessment

EAE was actively induced according to previously published protocols [17]. Emulsions for immunization were prepared by homogeneously combining complete Freund’s adjuvant (CFA), a generated mixture of incomplete Freund’s adjuvant (IFA) and *Mycobacterium tuberculosis* H37RA at 8 mg/mL (both from Difco Laboratories, Detroit, MI, USA), with equal volumes of myelin-derived peptide solution using glass syringe extrusion. Female SJL/J mice (8–10 weeks old) were immunized with 100 μg PLP_139–151_ (HCLGKWLGHPDKF) peptide (Peptide Synthesis Core Facility, German Cancer Research Center). Mice were shortly anaesthetized using 2–3 % (v/v) isoflurane (Baxter, Unterschleissheim, Germany) and 50 µL of the antigen/CFA emulsion was injected subcutaneously (s.c.) into two semi-lateral thoracic sites. Clinical signs were evaluated daily in a blinded fashion according to a standard EAE grading scale: 0, no signs; 1, limp tail or hind limb weakness; 2, limp tail and hind limb weakness or weakness of both hind limbs; 3, complete hind limb paralysis; 4, quadriplegia; and 5, moribund or death. Animals showing clinical signs in between these grades were scored intermediate using half increments [17]. Mice were euthanized by CO_2_ inhalation when reaching a score of 4.5 or at the end of the experiment.

### Experimental treatment design

Treatment of disease was performed during the first remission phase. Only those mice were included into the experimental groups which showed at least a score of 3 in the acute phase and had a score of 1 or 0 on the two consecutive days before cellular treatment. Dependent on the course of disease, therapy started between day 20 and 22 post immunization by injection of 2 × 10^7^ cells (suspended in 100 µL PBS w/o Ca^2+^/Mg^2+^) into the tail vein on each of three consecutive days. Administration of PBS into EAE mice served as control. Mice were assigned to the various treatment groups to obtain a similar distribution of mice within all groups regarding the course of the EAE disease as well as the current degree of clinical signs. One day prior to cell therapy, splenocytes (SPCs) were isolated from mice which had been immunized with the EAE-inducing peptide-CFA emulsion at the same time as those mice assigned to the different treatment groups. The majority of animals serving as cell donors showed clinical signs of disease of various degrees, whereas only very few did not exhibit any symptoms after EAE induction. Harvested SPCs were pooled before treatment. Donor mice with a similar distribution of clinical EAE scores were chosen for each of the three consecutive treatment days.

### Immunization with ovalbumin

After successful treatment of EAE mice with MIC_Cop_, the animals were immunized s.c. with 100 μL ovalbumin (OVA)/IFA emulsion (Hooke Laboratories, Lawrence, MA, USA) on day 55 and boosted on day 74 post EAE induction.

Naïve as well as PBS-treated EAE-induced mice were included as controls. Mice were sacrificed 33 or 37 days after the last OVA immunization. Anti-OVA antibody titers in serum and OVA-specific T-cell proliferation of lymph node cells and SPCs were analyzed.

### Preparation of serum and cells from peripheral lymphoid organs

After mice were sacrificed by CO_2_ inhalation, whole venous blood was collected and allowed to clot at room temperature. The clot was removed by centrifugation and serum was subsequently stored at −20 °C. Spleen and lymph nodes were retrieved, disintegrated mechanically and filtered through a 70-μm nylon Falcon^®^ cell strainer (Corning Life Sciences, Amsterdam, The Netherlands). After washing the cells with PBS (PromoCell, Heidelberg, Germany) lysis of erythrocytes was performed for SPCs by suspending the pelleted SPCs in 0.2 % NaCl for 30 s followed by two wash steps with culture medium, consisting of RPMI 1640 (PromoCell) supplemented with 10 % FCS (Lonza, Cologne, Germany), 100 U/mL Penicillin/100 μg/mL Streptomycin (PAA, Coelbe, Germany), 1 % l-glutamine (PAA) and 50 μM β-mercaptoethanol (Carl Roth, Karlsruhe, Germany).

### Generation of autoantigen-loaded splenocytes, treatment with MMC and UV/C irradiation

Freshly isolated SPCs (5 × 10^6^/mL in 12-well plates) were incubated o/n with or without 10 µg/mL glatiramer acetate (GA, Copaxone^®^, Cop; Teva Pharma, Kirchzarten, Germany) in culture medium. Cells were harvested on ice the next day and washed with culture medium. Afterwards the cells were either treated with mitomycin C (MMC; medac, Wedel, Germany), UV/C-irradiated or left untreated. For MMC treatment, 5 × 10^6^ SPCs/mL were incubated in 50 µg MMC/mL for 30 min in a humidified incubator (37 °C, 5 % CO_2_) and subsequently washed twice. UV/C irradiation was performed directly after antigen-loading (in 12-well-plates) in a Stratalinker 1800 device (Stratagene, Santa Clara, CA, USA) using 25 mJ/cm^2^. The cells were harvested on ice and washed twice.

### Isolation of regulatory T lymphocytes (Tregs)

Pooled single cell suspensions of SPCs and lymph node cells (LNCs), herein referred to as peripheral blood mononuclear cells (PBMCs) were obtained from EAE mice 2 weeks after immunosuppressive therapy with MIC_Cop_ as described above. Regulatory CD4^+^CD25^+^ T lymphocytes (Tregs) were purified by consecutive negative isolation of CD4^+^ cells and positive selection of CD25^+^ lymphocytes using a MACS^®^ magnetic microbead kit as specified by the manufacturer (Miltenyi Biotec, Bergisch Gladbach, Germany), usually resulting in a purity above 90 % of the CD4^+^CD25^+^ cell population. Importantly, initial flow cytometric examination revealed that nearly all (>95 %) CD4^+^CD25^+^ lymphocytes also expressed the transcription factor FoxP3.

### Cellular assays, cytokine expression and antibody detection

For proliferation and cytokine secretion assays, SPCs and LNCs were isolated from treated mice. Cells were seeded in U-bottom 96-well plates (Greiner, Frickenhausen, Germany) at 2 × 10^5^ cells/well in 200 µL culture medium and stimulated with either phytohemagglutinin (PHA) (Remel, Lenexa, USA) at 45 µg/mL or reactivated with 5–20 µg/mL PLP_139–151_, Cop or 10 µg/mL OVA (chicken; Sigma-Aldrich, Taufkirchen, Germany). For analyses of T-cell proliferation, after 48 or 72 h, cells were pulsed with [^3^H]-thymidine (Hartmann Analytic, Braunschweig, Germany) at 1 µCi/well for further 18 h, harvested onto filter plates and [^3^H]-thymidine incorporation was measured in an automated β-counter (Inotech Biosystems, Rockville, MD, USA).

For assessment of cytokine expression, supernatants were obtained after 72 h of culture. The cytokines IL-2, IL-4, IL-6, IL-10, IL-12, IL-17, interferon-γ (IFN-γ), and transforming growth factor-β (TGF-β) and tumor necrosis factor-α (TNF-α) were quantified by enzyme-linked immunosorbent assay (ELISA) using commercial kits according to the manufacturer’s instructions (eBioscience, Frankfurt, Germany).

For cellular inhibition assays, 5 × 10^4^ PBMCs per well were stimulated with PHA (45 µg/mL) and cocultured with 1 × 10^4^ isolated CD4^+^CD25^+^ Tregs (5:1 ratio) for 72 h followed by incubation with [^3^H]-thymidine. Cell populations with and without PHA stimulus served as controls.

In addition, isolated CD4^+^CD25^+^ Tregs as well as conventional CD4^+^ effector T lymphocytes (4 × 10^5^/well) were stimulated for 48 h with plate-bound hamster-anti-mouse-CD3 mAb (10 µg/mL, clone 145-2C11; BD Biosciences, Heidelberg, Germany). Supernatants were obtained and concentration of IL-10 was determined by ELISA (eBioscience).

Anti-OVA antibodies (Abs) were detected in serum from treated and OVA-immunized mice using a mouse anti-OVA ELISA kit according to the manufacturer’s instruction (Hooke Laboratories). Optical density of assay samples was measured photometrically in a microplate reader (Tecan, Maennedorf, Switzerland) and cytokine concentrations and anti-OVA Ab titers were calculated by means of the respective standards.

### Flow cytometric analysis of cellular markers and viability

Approximately 1 × 10^6^ SPCs were washed in PBS and blocked for 10 min on ice with 1 μg mouse BD Fc block CD16/CD32 (BD Biosciences) in 100 µL FACS-buffer, PBS containing 0.1 % bovine serum albumin (BSA; Carl Roth), and then incubated with PE-conjugated anti-mouse-CD4 mAb (clone RM4–5, rat IgG2a, κ) as well as FITC-conjugated anti-mouse CD25 mAb (clone 3C7, rat IgG2b, κ) or their corresponding isotype controls (BD Biosciences) for 15 min at 4 °C in the dark. For further intracellular detection of the transcription factor Foxp3, cells were fixed, permeabilized and stained with rat-anti-FoxP3 antibody (clone FJK-16 s; eBioscience). Cell viability was determined with 7-aminoactinomycin D (7-AAD) and Annexin V (BD Biosciences). Cells were examined using a FACSCalibur™ flow cytometer (BD Biosciences) and data were analyzed with CellQuest™ Pro software (BD Biosciences).

### Histological examination

The spine was dissected, immediately embedded into Tissue-Tek^®^ (Sakura Finetek, Zoeterwoude, The Netherlands) and frozen on dry ice. Specimens were stored at −80 °C until further analysis. Seven µm-thick sections of the lumbar region were cut on a cryostat, air-dried and post-fixed in acetone. Sections were blocked with 5 % BSA (PAA) and 3 % mouse serum (Sigma-Aldrich) in PBS for 30 min and then incubated with the primary antibody directed against FoxP3 (clone FJK-16 s; eBioscience) diluted 1:4800 in 1 % BSA solution at 4 °C overnight. The next day, sections were incubated with biotin-conjugated rabbit anti-rat IgG (1:400) (Dako, Hamburg, Germany) in 1 % BSA solution at RT for 40 min followed by Neutravidin-Dylight549 (Thermo Scientific, Waltham, MA, USA) at 1:500 dilution in washing solution (TBS + 0.05 % Tween^®^ 20). Counterstaining of cellular nuclei was performed by incubation with Hoechst 33,342 (1:1000 in washing solution; Thermo Scientific). Sections were analyzed on a Zeiss Axioskop 50 epifluorescence microscope using Carl Zeiss Plan-NEOFLUAR 109/0.30 and 409/1.30 objectives and Carl Zeiss filter sets No. 1 (excitation BP 365/12, emission LP 397) and No. 15 (excitation BP 546/12, emission 590 nm) for detection of fluorescence. Digital images were acquired using a Leica DFC350FX camera and software.

### Biodistribution studies for in vivo tracking of ^111^Indium-labelled MICs

MIC_Cop_ (4–5 × 10^7^) from naïve SJL/J mice were generated as described and labeled with 20 MBq ^111^Indium (In)-oxine (Mallinckrodt Pharmaceuticals, Dublin, Ireland) in PBS for 15 min at room temperature. After removal of free ^111^In-oxine by washing with 50 mL PBS, the cells were resuspended in 130 µL PBS and 50 µL of cell suspension was injected into the tail vein of recipient mice. Twenty-four hours later, mice were anaesthetized with 1 % (v/v) sevoflurane (Baxter) and, after blood samples had been taken from the vena cava, perfusion with Ringer’s solution (B.Braun, Melsungen, Germany) was conducted until blood was completely washed out from the organ system. Tissue samples of organs were harvested and weighed. Radioactivity of the specimen was measured along with 10 µL-aliquots (n = 3) of the injected suspension in a γ-counter (LB951G, Berthold Technologies, Bad Wildbad, Germany). For each sample, the activity of the ^111^In-tracer in 1 g of tissue was calculated in relation to the originally injected total dose (% ID/g).

### Statistical analysis

Statistical analysis was performed using GraphPad Prism 5.0 (GraphPad Software, La Jolla, CA, USA) and IBM SPSS predictive analytics software (IBM, Armonk, NY, USA). Results were assessed by applying Student’s *t* test or ANOVA when normal Gaussian distribution was given. In contrast, the nonparametric Mann–Whitney *U*- or Fisher’s exact test was used for comparison of distribution-free data sets, not covered by normal distribution. The limit of statistical significance was *p* ≤ 0.05 whereas a *p* value of < 0.05 was considered to be significant (*), a *p* value of < 0.01 highly significant (**) and a *p* value of < 0.001 extremely significant (***).

## Results

### Mitomycin-induced cells loaded with Copaxone^®^ (MIC_Cop_) reduce relapses of ongoing remitting-relapsing EAE

**Fig. 1:**
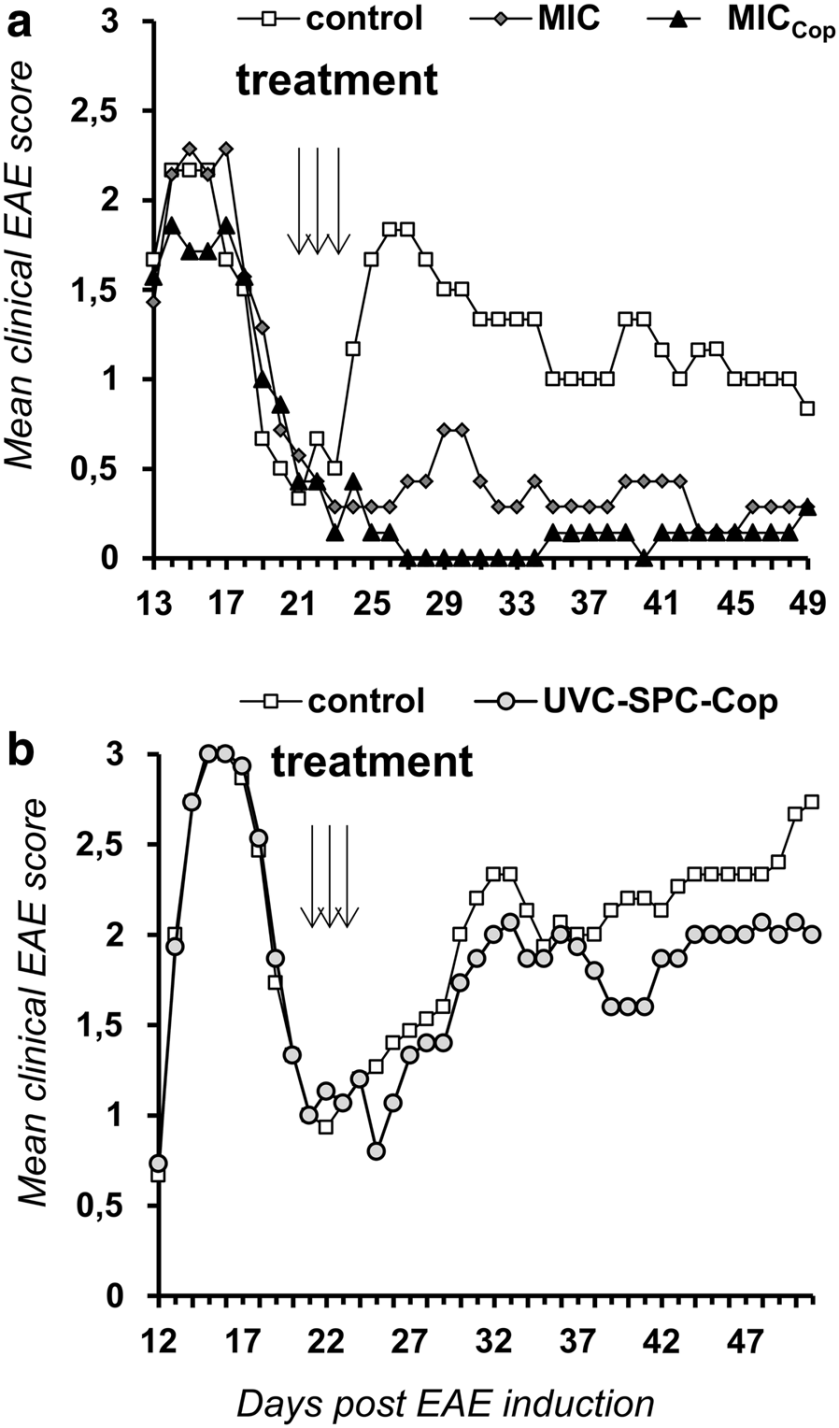
MIC_Cop_ reduce relapses of ongoing disease in remitting-relapsing EAE. A relapsing-remitting EAE was induced in female SJL/J mice at day 0 with injection of proteolipid peptide PLP_139–151_ in complete Freund’s adjuvant. Clinical signs were evaluated daily. For cellular treatment, SPCs were isolated from EAE animals, cultured in vitro o/n with or without 10 µg/mL glatiramer acetate (GA, Copaxone^®^, Cop). On the day of cell therapy, splenocytes were either treated with 50 µg/mL MMC or UV/C-irradiated with 25 mJ/cm^2^. During first remission, animals of treatment groups received 2 × 10^7^ cells on each of three consecutive days (days 21–23): **a** MMC-treated SPCs (MICs; n = 7, *dark grey diamonds*), Cop-loaded and MMC-treated SPCs (MIC_Cop_; n = 7, *black triangles*) or PBS (control; n = 6, *white squares*); **b** Cop-loaded and UV/C-irradiated SPCs (UVC-SPC_Cop_; n = 15, *grey circles*) or PBS (control; n = 15, *white squares*). Each *panel* shows the time course of the mean EAE score for the respective treatment group

We generated MIC_Cop_ cells by loading syngeneic spleen cells (SPCs) in vitro with Cop and incubating them with MMC. In a series of experiments (Fig. 1a) the effect of MIC_Cop_ was analyzed in a relapsing-remitting form of EAE. The disease was induced via PLP in SJL/J mice and 2 weeks later the peak of paralysis was achieved. One week later the animals entered into remission. SPCs of syngeneic EAE animals were collected and used for preparation of MIC_Cop_. The cells were injected on three consecutive days. In contrast to untreated controls (white squares), treated animals showed nearly no relapses (black triangles). Interestingly, when injecting SPCs incubated with MMC only (MIC), a similar albeit weaker inhibition was noted (dark grey diamonds).

For clinical application one important objective is the prevention of further relapses. Table 1 shows that MIC_Cop_ therapy reduces the number of relapses from 70.5 % (control) to 16.7 % (*p* < 0.0001). The duration of relapse is also significantly reduced (median duration from 20 days in the control to 4 days in the MIC_Cop_ group, *p* = 0.017). In contrast, MIC therapy neither reduced the number of relapses (*p* = 0.66) nor their duration (*p* = 0.063) when compared to PBS-treated controls. Its suppressive effect (number of relapses) was significantly weaker than in the MIC_Cop_ group (*p* = 0.045) but was not different in respect to the duration of relapse (*p* = 0.476).

**Table 1:** Incidence and duration of paralytic relapses in EAE mice after cell treatment

Treatment group	Incidence of relapse^a, b^	Relapse rate (%)	Duration of relapse^c^		
			Median	Mean ± SD	CI (95 %)
Control (PBS)	31 (44)	70.5	20.0	17.0 ± 9.6	13.6–20.4
MIC_Cop_	5 (30)	16.7	4.0	6.6 ± 5,0	2.2–11.0
MIC	4 (7)	57.1	6.5	9.50 ± 8.4	1.3–17.7
SPC_Cop_	8 (15)	53.3	13.5	14.5 ± 6,6	9.9–19.1
UVC-SPC_Cop_	15 (15)	100	24.0	19.6 ± 7.7	15.7–23.5

Treatment groups were compared using Fisher’s exact test (incidence of relapse) and One-tail Mann–Whitney *U* test (duration of relapse): * *p* < 0.05, ** *p* < 0.01, *** *p* < 0.001

*CI* confidence interval; *EAE* experimental autoimmune encephalomyelitis; *MIC* mitomycin C-induced cells; *MIC*_*Cop*_ Copaxone^®^-loaded mitomycin C-induced cells; *PBS* phosphate-buffered saline; *SD* standard deviation; *SPC*_*Cop*_ Copaxone^®^-loaded splenocytes; *UVC-SPC*_*Cop*_ Copaxone^®^-loaded ultraviolett C-irradiated splenocytes

^a^Incidence of relapse: number of mice with relapse (total number of animals) in treatment group

^b^ *Incidence of relapse* MIC_Cop_ vs. control, *** *p* < 0.0001; MIC vs. control, *p* = 0.66; SPC_Cop_ vs. control, * *p* = 0.026; UVC-SPC_Cop_ vs. control, * *p* = 0.034; MIC_Cop_ vs. MIC,* *p* = 0.045; MIC_Cop_ vs. SPC_Cop_, *** *p* < 0.0001; MIC_Cop_ vs. UVC-SPC_Cop_, * *p* = 0.016; MIC vs. SPC_Cop_, *p* = 1.00; MIC vs. UVC-SPC_Cop_, * *p* = 0.022; SPC_Cop_ vs. UVC-SPC_Cop_, ** *p* = 0.006

^c^ *Duration of relapse* MIC_Cop_ vs. control, * *p* = 0.017; MIC vs. control, *p* = 0.063; SPC_Cop_ vs. control, *p* = 0.266; UVC-SPC_Cop_ vs. control, *p* = 0.755; MIC_Cop_ vs. MIC, *p* = 0.476; MIC_Cop_ vs. SPC_Cop_, * *p* = 0.024; MIC_Cop_ vs. UVC-SPC_Cop_, ** *p* = 0.005; MIC vs. SPC_Cop_, *p* = 0.107; MIC vs. UVC-SPC_Cop_, * *p* = 0.026; UVC-SPC_Cop_ vs. SPC_Cop_, *p* = 0.945

It is known that Cop *per se* already inhibits EAE [18, 19]. Therefore, we addressed the question whether SPCs incubated with Cop only (without MMC) also have an inhibitory action. The findings demonstrated (Table 1) that they significantly reduce the rate (*p* = 0.026) but not the duration of relapse (*p* = 0.266). In both cases the suppressive effect was significantly weaker than that of MIC_Cop_ (*p* < 0.0001 and *p* = 0.024, respectively).

### UV-induced apoptotic cells loaded with Copaxone^®^ do not prevent relapses of ongoing EAE

MMC is a chemotherapeutic drug, which induces apoptosis in tumor cells [20]. Our studies showed that the same happens with murine SPCs when incubated with MMC (% apoptotic cells 3, 6, 12, 18, 24, and 30 h after incubation with MMC: 1.8, 3.6, 12.2, 46.1, 67.3 and 82.9). This raised the question whether apoptosis might be responsible for the observed immunosuppression. In a follow-up experiment, apoptotic cells were generated by UV/C irradiation. An equivalent number of apoptotic cells (corresponding to that obtained via MMC) loaded with Cop was injected into sick mice. In contrast to MIC_Cop_ treatment, no inhibition of disease was noted (Fig. 1b grey circles). On the contrary, UVC-SPC_Cop_ cells increased the number of relapses from 70.5 % (control) to 100 % (*p* = 0.034) and their duration (median) from 20 days (control) to 24 days (*p* = 0.755) (Table 1).

### Suppression induced by MIC_Cop_ therapy is autoantigen-specific

A series of therapeutic agents can control EAE in mice or MS in humans, but many of them exert an unspecific immunosuppressive action. We addressed the question whether MIC_Cop_ therapy suppresses only the deleterious immune response against the central nervous system (CNS), while preserving other immune responses.

**Fig. 2:**
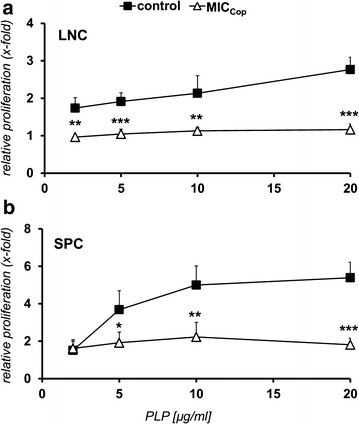
MIC_Cop_ therapy suppresses the PLP-specific T-cell response. SJL/J mice were induced to exhibit clinical signs of EAE. In their first phase of remission, on days 21–23 post disease induction, mice were treated with MIC_Cop_ (*white triangles*) or PBS (control, *black squares*). LNCs (**a**) and SPCs (**b**) of MIC_Cop_-treated (n = 8) and control (n = 9) EAE mice were harvested on day 49 after disease induction. Cells of each treatment group were restimulated in vitro with PLP_139–151_ for 48 h at the indicated concentrations (abscissa). Proliferation was determined by [^3^H]-thymidine incorporation and is displayed as x-fold increase in relation to unstimulated cells (ordinate). The mean ± standard error of the mean (SEM) of a sixtuplicate setup was calculated. One representative of three independent experiments is shown. Unpaired Student’s *t* test was performed for comparison of the treatment groups (*p < 0.05; **p < 0.01; ***p < 0.001)

**Fig. 3:**
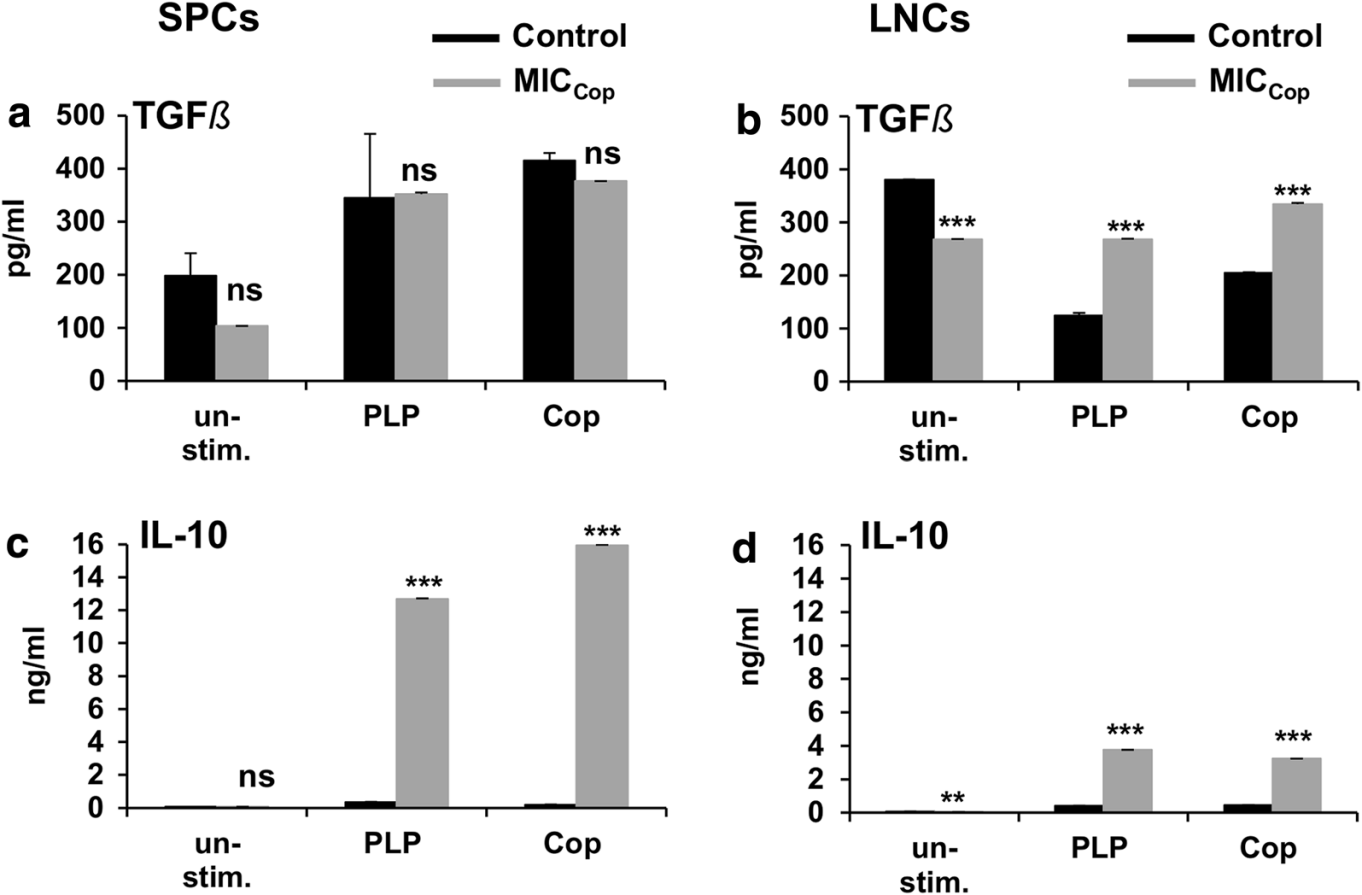
Cytokine expression of peripheral lymphocytes from MIC_Cop_-treated EAE mice after in vitro-restimulation with PLP and Copaxone^®^. Splenocytes (SPCs; *left column*) and lymph node cells (LNCs; *right column*) were isolated from PBS- (control, *black*, n = 9) and MIC_Cop_-treated (MIC_Cop_, *grey*, n = 8) EAE mice and restimulated in vitro with 20 µg/mL PLP_139-151_ or Copaxone^®^ (Cop). After 72 and 96 h, supernatants were collected and concentrations of TGF-β (**a** and **b**) and IL-10 (**c** and **d**) were determined by ELISA. Unstimulated cells represent basic cytokine expression. The mean ± standard error of the mean (SEM) was calculated for values measured in duplicate. Treatment groups were compared using unpaired Student’s *t* test (***p < 0.001; **p < 0.01; *p < 0.05; ns, not significant)

In one experiment lymphocytes derived from lymph nodes or spleens of EAE mice having received MIC_Cop_- or control-therapy were re-stimulated in vitro with various concentrations of PLP—the disease-inducing autoantigen. The experiment depicted in Fig. 2 shows that peripheral T lymphocytes of MIC_Cop_-treated animals (white triangles) did not respond to PLP, in contrast to those of PBS-treated control mice (black squares). This demonstrates suppression of the autoantigen-specific T-cell response. No response to MBP_84-104_ and MOG_92-106_ was detected even in untreated EAE mice, showing that epitope-spreading from PLP to MBP and MOG did not take place during progression of disease (data not shown). Next, we wanted to see what the cytokine pattern of these non-responding lymphocytes derived from MIC_Cop_-treated animals looks like when the cells are exposed to PLP or Cop. As shown in the experiment presented in Fig. 3a–d, SPCs and LNCs produce more IL-10, whereas LNCs also secrete more TGF-β than cells of untreated EAE animals. No differences were noted regarding the expression of IL-2, IL-4, IL-6, IL-12, IFN-γ, TNF-α and only partially increased expression of IL-17 was observed (data not shown).

**Fig. 4:**
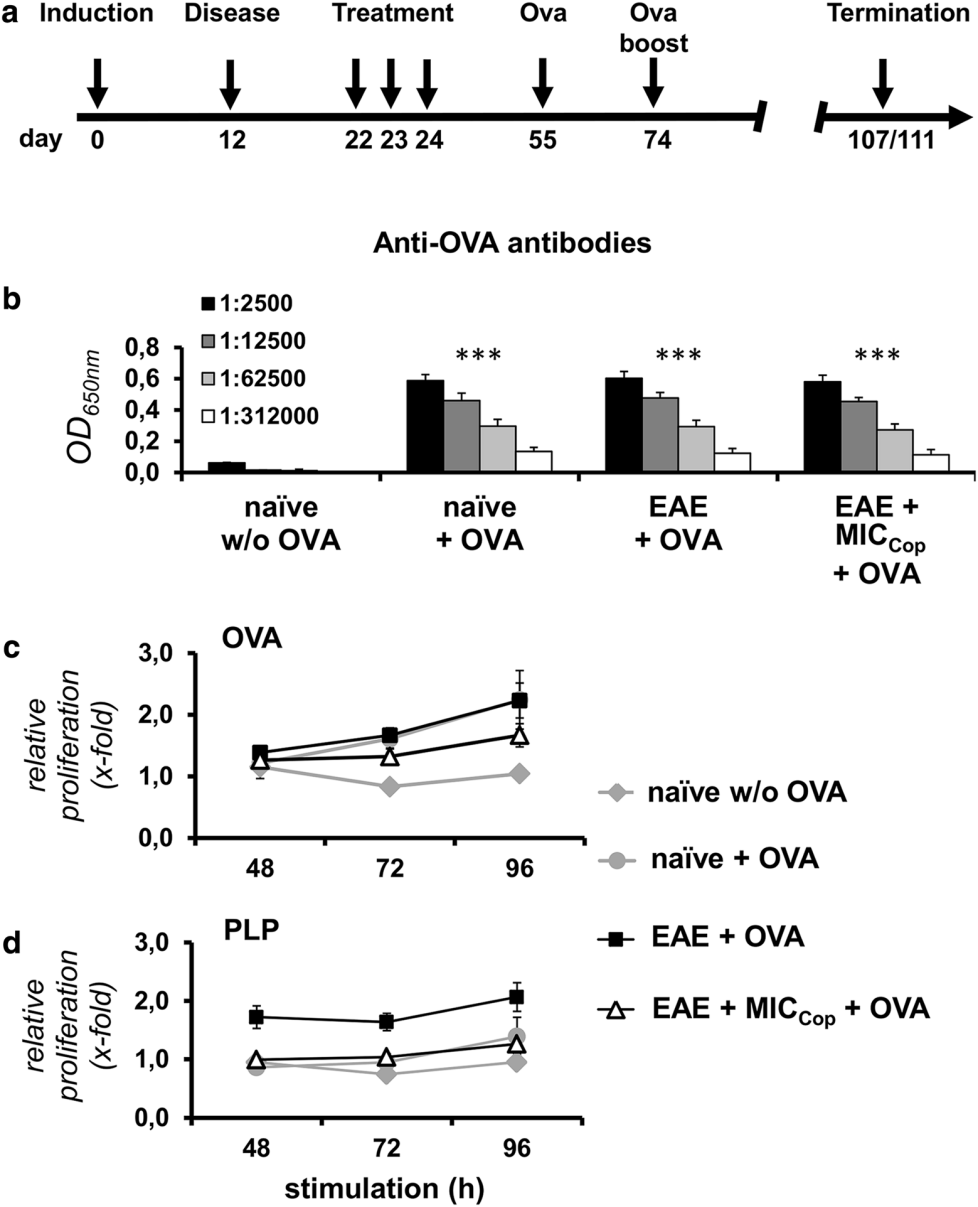
MIC_Cop_ therapy does not suppress the immune response to foreign antigens. **a** MIC_Cop_- (n = 8) or PBS-treated (n = 8) EAE mice were immunized with ovalbumin (OVA) on day 55 and 74 after disease induction. Additional controls comprised healthy animals with OVA (naïve + OVA, n = 4) and w/o OVA immunization (naïve w/o OVA, n = 2). Animals were sacrificed on days 107 and 111. Serum was obtained and peripheral mononuclear cells from lymph nodes (LNCs) were isolated. **b** Anti-OVA antibodies were detected by ELISA in sera of single animals diluted 2500 to 312,000-fold. The graph shows mean values ± standard deviation (SD). For comparison of the different groups with the naïve w/o OVA control the unpaired Student’s *t* test was used (***p < 0.001). **c** and **d** OVA- and PLP-specific T-cell proliferation of LNCs harvested from single animals was assessed by in vitro restimulation with OVA protein (**c**) and PLP_139–151_ (**d**). Proliferation was determined after 48, 72 and 96 h by [^3^H]-thymidine incorporation and is indicated as x-fold increase in relation to unstimulated cells (ordinate). Shown is the mean ± standard error of the mean (SEM) of every group. The differences of T-cell responses towards OVA among the groups naïve + OVA, EAE + OVA and EAE + MICcop + OVA were statistically not significant ( **c**) whereas the proliferative response of MIC_Cop_-treated mice upon PLP stimulation was still suppressed after OVA immunization ( **d**; EAE + MIC_COP_ + OVA vs. EAE + OVA after 48 h: *p* < 0.01; 72 h: *p* < 0.05; 96 h: *p* < 0.01). As control, LNC proliferation against OVA of naïve mice immunized with OVA was significantly stronger than that of naïve mice without OVA treatment (96 h: *p* < 0.01). Two-way-ANOVA test with Bonferroni correction was used

In a second series of experiments the immune response to foreign antigens of EAE mice treated with MIC_Cop_ was studied. The animals were immunized with ovalbumin (OVA) (Fig. 4a). As controls served PBS-treated EAE- or naïve mice immunized with OVA. Antibody as well as T-cell responses against OVA were measured. As shown in Fig. 4b, the antibody response of diseased animals was not influenced by MIC_Cop_-treatment. Regarding T cells, although the MIC_Cop_-treatment seemed to slightly inhibit the response to OVA, the suppression was statistically not significant (Fig. 4c). The immune response of MIC_Cop_-treated animals to PLP, however, was still absent after OVA immunization (Fig. 4d; relative proliferation of EAE + OVA vs. EAE + MIC_Cop_ + OVA after 48 h, **p < 0.01, 72 h, *p < 0.05 and 96 h, **p < 0.01). Taken together, these findings show that MIC_Cop_-treated EAE mice can develop an immune response to third party antigens, whereas the response to the disease-inducing autoantigen remains suppressed.

### MIC_Cop_ cells migrate into the spleen and other organs

**Fig. 5:**
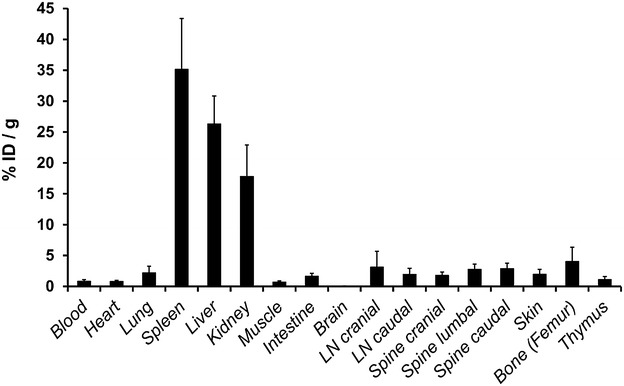
Tracking of injected MIC_Cop_ cells in mice. SPCs from naïve SJL/J mice were loaded with Copaxone^®^ (Cop) o/n and then treated with 50 µg/mL MMC (MIC_Cop_). 2 × 10^7^ MIC_Cop_ were labeled with 20 MBq ^111^Indium (In)-oxine and injected into the tail vein of the recipient mouse. Twenty-four hours later the animals were euthanized, thoroughly perfused with Ringer’s solution and single organs were harvested and weighed. The activity of tissue samples as well as of an aliquot of the administered labeled cell suspension was determined for each animal in a γ-counter and calculated as  % of total injected dose (ID) per gram tissue. Shown is the in vivo distribution of activity in the indicated organs of four mice depicted as mean ± SD (*LN* lymph node)

Our previous findings showed that allogeneic MICs injected into rats migrate mainly into the spleen, leading to a locally increased infiltration with CD4^+^CD25^+^FoxP3^+^ Tregs [21]. This prompted us to analyze whether syngeneic MIC_Cop_ cells labeled with ^111^Indium have a similar fate when injected into mice. As shown in Fig. 5 this was the case.

### Treatment of EAE mice with MIC_Cop_ increases the CD4^+^CD25^+^FoxP3^+^ cell infiltration of peripheral lymphoid organs and the central nervous system

**Fig. 6:**
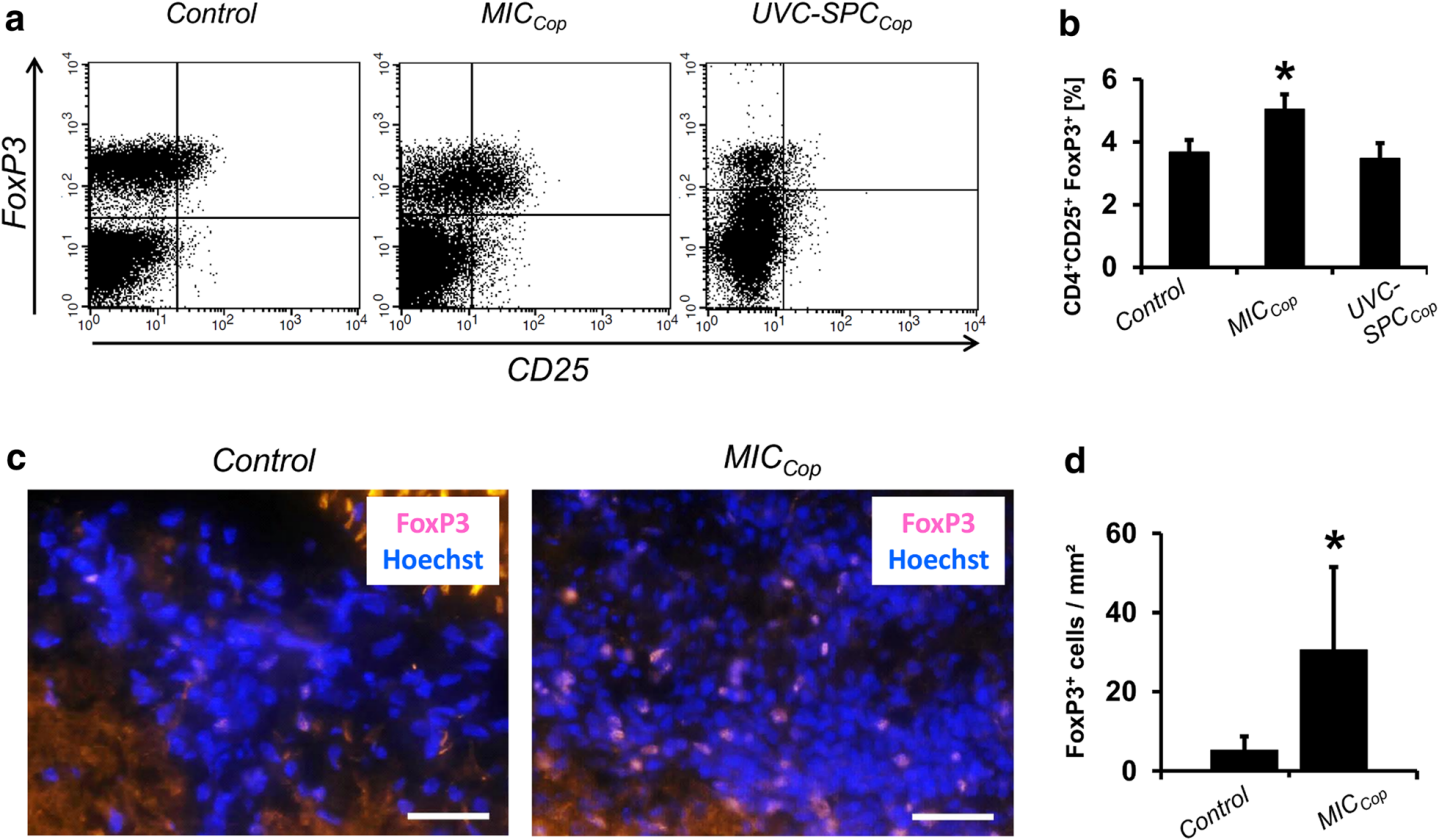
Treatment of EAE mice with MIC_Cop_ increases CD4^+^CD25^+^FoxP3^+^ cell infiltration of peripheral lymphoid organs and the central nervous system. The infiltration with CD4^+^CD25^+^FoxP3^+^ regulatory T cells (Tregs) of peripheral lymphoid organs (**a** and **b**) and the central nervous system (**c** and **d**) of cell-treated and control EAE mice was determined with FACS analysis in SPCs and immunohistochemistry in spinal cord sections, respectively. **a** For FACS analysis SPCs of animals treated with PBS (control), MIC_Cop_ and UVC-SPC_Cop_ were isolated 4 weeks after cell therapy and stained with fluorescence-labeled antibodies and the corresponding isotype controls. One representative FACS-plot is shown for each group representing the expression of CD25 and FoxP3 in CD4^+^ cells. **b** Tregs within SPCs of each treatment group were quantified and are depicted as percentage of CD4^+^CD25^+^FoxP3^+^ T cells within the CD4^+^ T-cell population (mean value ± SEM; n = 8 per group). Data were statistically compared with the unpaired Student’s *t* test (*p < 0.05). **c** Immunohistochemistry of the lumbar spinal cord for evaluation of infiltrating Tregs was performed on snap-frozen tissue of PBS- and MIC_Cop_-treated animals. After incubation of tissue sections with anti-FoxP3-antibody, Tregs were visualized with biotin-conjugated rabbit anti-rat IgG and Neutravidin-Dylight549. Cellular nuclei were counterstained with Hoechst 33,342 (*blue*). Digital fluorescence images were obtained at ×40 magnification and the number of infiltrated Tregs (*pink*) was determined. Scale bars depict 50 µm. **d** Statistical analysis of evaluated sections based on values from single animals (n = 8 for each group) was performed using Mann–Whitney *U* test (*p < 0.05). Mean values of FoxP3^+^ cells per mm^2^ ± SEM are presented

Spleens of MIC_Cop_-treated EAE mice were collected, cells prepared and analyzed by flow cytometry. As shown in Fig. 6a (middle panel) MIC_Cop_ treatment led to an increased number of CD4^+^CD25^+^FoxP3^+^ cells as compared to treatment with UV-induced apoptotic cells (right panel) or PBS (left panel). Statistics are shown in panel B.

Next, we addressed the question whether MIC_Cop_ treatment increases the infiltration of the CNS—the target of the autoimmune attack—with CD4^+^CD25^+^FoxP3^+^cells. The results show that this is indeed the case. Panel C shows representative histological sections of the spinal cord from MIC_Cop_-treated and control mice, and panel D the statistical representation of these findings.

### CD4^+^CD25^+^FoxP3^+^ cells exhibit an immunosuppressive activity

**Fig. 7:**
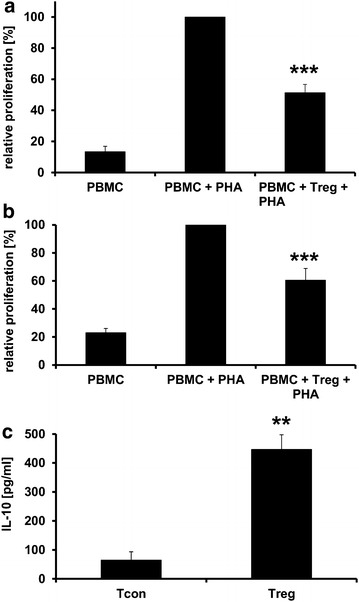
Characterization of regulatory CD4^+^CD25^+^FoxP3^+^ T cells of MIC_Cop_-treated EAE mice. CD4^+^CD25^+^ regulatory T lymphocytes (Tregs) of MIC_Cop_-treated SJL/J mice were separated from LNCs and SPCs (= PBMCs) 2–3 weeks after cellular treatment via a microbead-based MACS protocol. For each animal Tregs from LNCs and SPCs were pooled. **a** PBMCs of PBS- (control, n = 12) or **b** MIC_Cop_-treated (n = 12) EAE animals were stimulated with phytohemagglutinin (PHA) and co-incubated with Tregs derived from single animals treated with MIC_Cop_ (*black bars*) at a ratio of 5:1 (n = 12). Proliferation was determined by [^3^H]-thymidine incorporation. Positive control was PHA-induced T-cell proliferation of PBMCs only (100 %). Graphs show the mean relative proliferation rate (%) ± SEM in relation to the positive control. For statistical analysis One-way-ANOVA with Bonferroni correction was used (***p < 0.001; n = 12). **c** Tregs derived from SPCs and LNCs from two animals were pooled (in total n = 6), seeded (4 × 10^5^/200 µL/well) and stimulated with plate-bound anti-CD3 monoclonal antibody for 48 h. IL-10 secretion was analyzed by ELISA. Stimulated conventional CD4^+^ T cells from the same animals served as control. Data are mean ± SEM (Student´s *t* test; **p < 0.01)

We examined whether the infiltrating CD4^+^CD25^+^FoxP3^+^ cells have suppressive activity. CD4^+^CD25^+^FoxP3^+^ cells were obtained by MACS separation from spleen and lymph nodes of MIC_Cop_-treated animals. Syngeneic T cells from PBS-treated EAE animals were stimulated with PHA and CD4^+^CD25^+^FoxP3^+^ cells added to the culture. Figure 7a, b shows that CD4^+^CD25^+^FoxP3^+^ cells inhibit the polyclonal T-cell response, thus proving their suppressive potential. Thereafter, IL-10 cytokine expression of CD4^+^CD25^+^FoxP3^+^ cells was analyzed (Fig. 7c). The result shows an increased production of this immunomodulatory cytokine as compared to conventional T cells (Tcons).

## Discussion

Broad immunosuppression has been the standard therapy for autoimmune diseases during the past half century. The main drawback of this therapy is the lack of distinction between harmful responses to self-antigens and useful responses to foreign antigens. Recently, a series of novel therapeutic options aiming at re-establishing specific tolerance of the derailed autoimmune response have been conceived (reviewed by [22]).

Apart from chemical or biologic therapeutics, such as monoclonal antibodies, four main cell-based therapies for the induction of tolerance in autoimmune diseases are currently under investigation: hematopoietic chimerism, mesenchymal stromal cells (MSCs), Tregs and DCs [22]. Establishing a hematopoietic chimerism, although effective in some cases, remains a quite invasive intervention, difficult to implement into clinical routine [23]. Although treatment with MSCs initially seemed to be safe and possible improvement of disease was noted [24–28], recent observations showing that MSCs might differentiate into sarcoma cells [29] or protect breast cancer cells through Tregs [30] question their application. Ex vivo expansion of Tregs also poses problems when it comes to clinical application, such as the inclusion of harmful effector T cells [31] or the possible abolition of the suppressive phenotype following injection into patients. Moreover, as shown recently, Tregs might promote metastatic spread of mammary cancer cells [32]. A series of studies showed that tolerogenic DCs (tolDCs) can be used for controlling autoimmune diseases in animal models [10]. However, their use for the treatment of autoimmune disorders in humans is still in its infancy [10, 33]. The main challenge in bringing tolDCs into the clinic is the requirement to preserve their tolerogenic property upon transfusion into the patient.

Major drawbacks of almost all therapies are the laborious and time-consuming process of cell production as well as the high costs. In the present article, we describe a therapy which suppresses specifically the harmful immune response against the CNS and is based on suppressive cells which can be easily and quickly generated. Moreover, the cells are stable and can be prepared in large amounts at rather moderate costs.

An elegant cell therapeutic approach conceptually close to ours, which has already been tested in phase-1 trial, is the use of autologous peripheral blood mononuclear cells chemically coupled with seven peptides derived from myelin proteins [34]. This model is based on the pioneering work of S.D. Miller in animals [35]. Previous observations demonstrated that MBP-like peptides can lead to an exacerbation of the disease [36]. What distinguishes our model from the one above is, among others, the lack of use of potentially harmful autoantigens. Instead, Copaxone^®^—an approved MS-protecting drug—is used. A second component of our cell therapeutic is MMC—a well-known drug, which has been used for decades in cancer patients [37].

It is known that GA competes with MBP for binding to MHC-II on antigen-presenting cells and for its recognition by specific T cells [13]. GA also displaces PLP from the MHC binding site, inhibits PLP-specific T-cell responses and PLP-induced EAE [14, 38]. When preparing MIC_Cop_ we loaded the cells with Cop. Therefore, the question must be addressed whether MIC_Cop_ cells owe their therapeutic effect to the injected dose of Cop. What argues against this reasoning is the finding that untreated or UV-treated spleen cells loaded with this drug did not reduce the rate of relapses. UVC-SPC_Cop_ even enhanced the course of disease. Moreover, the dose of Cop used for preparing MIC_Cop_ was 0.040 mg per 2 × 10^7^ cells (number of injected cells), of which only a small fraction was taken up by the cells. Even if the whole amount of Cop would have been loaded onto cells, it would have been far lower than the therapeutic dose which lies in the range of 2 mg/mouse [38].

MMC is a chemotherapeutic agent and as such might induce side effects in patients. Therefore, the question must be addressed whether the amount of MMC contained in the MIC cell preparation might cause harm to patients. The therapeutic dose of MMC is 10–20 mg/m^2^. This would amount to 63–126 µg per mouse. Upon incubation of cells and subsequent washing, undetectable concentrations of MMC were noted (detection limit 0.13 µg/ml). No side effects are expected under these conditions. Another critical point might be the possible malignant transformation of cells after MMC-treatment. MIC cells, however, cannot further divide and enter into apoptosis. Both preclude side effects upon injection into the patient.

Previous studies showed that apoptotic cells might either stimulate or suppress the immune response, their regulatory effect depending on a series of factors, notably on the type of immunologically active molecules co-expressed with apoptosis [20]. Because MMC induces apoptosis in murine SPCs, this might provide an explanation for the immunosuppressive activity of MIC_Cop_. However, when injecting Cop-loaded SPCs rendered apoptotic by UV irradiation, no inhibition of disease was noted. A similar observation was made in our rat heart allograft model upon treatment of recipients with apoptotic donor cells [21]. Because apoptosis is an irreversible process, administration of MIC_Cop_ cells, which are underway to apoptosis, would preclude their return to a stimulatory status in a clinical setting.

When applied to MS patients, autologous cells can be harvested by cytapheresis, loaded with Cop and treated with MMC. To mimic this process in the present experimental study, SPCs of syngeneic sick mice were used for preparation of MICs. Interestingly, cells treated with MMC (without Cop or PLP) already showed a certain inhibitory effect. It is known that antigen-presenting cells from peripheral lymphoid organs of MS patients carry brain autoantigens [39, 40]. The same applies to antigen-presenting cells of EAE animals [39]. This observation might explain why treatment with MMC (without additional loading with Cop as a surrogate of autoantigen) confers suppressive properties. If MMC-treated cells without Cop already have a suppressive activity, the question must be addressed, why they should be loaded with Cop. When evaluating the effectiveness of MIC therapy, three parameters must be taken into account: the degree of paralysis, the number of animals suffering relapses as well as the duration of relapses. Regarding the mean degree and duration of paralysis, the difference between MIC and MIC_Cop_ was apparently modest. However, when considering the number of mice with paralytic attacks, the difference between MIC and MIC_Cop_ becomes evident.

Along with the control of relapses, induction of antigen-specific suppression is the most relevant property of MIC_Cop_ cell therapy. Treated mice responded normally to OVA and even to myelin autoantigens such as MBP or MOG. In a clinical setting this would allow to control the inflammatory process in the brain without affecting the remaining immune response. The question arose why the immune response to PLP but not to MBP and MOG was suppressed by MIC_Cop_. As already mentioned, Cop has the ability to interact with PLP-specific T cells [14]. When injecting MIC_Cop_ into EAE mice already sensitized to PLP (but not to MBP and MOG) the administered cells would be recognized first by highly affine PLP-specific T cells. This provides an explanation for the suppressed brain-damaging immune response against PLP.

While the mechanism of immunosuppression induced by MIC_Cop_ has not been exhaustively clarified, a couple of observations point to an involvement of Tregs in mediation of suppression. Their number increased in the spleen and CNS following injection of MIC_Cop_. We speculate that upon injection, MIC_Cop_ migrate into peripheral lymphoid organs, where they convert conventional PLP-specific T cells into CD4^+^CD25^+^FoxP3^+^ Tregs, which then migrate into the CNS. There, as well as in lymphoid organs, they produce suppressive cytokines, such as IL-10, unspecifically inhibiting the local immune reaction. The latter point is supported by our observation that CD4^+^CD25^+^FoxP3^+^ Tregs suppress the polyclonal T-cell response in vitro. Similar Tregs were also found in untreated EAE animals as a general regulatory mechanism of the immune response [41, 42]. However, their number was significantly lower than in treated mice. It seems that in untreated animals deleterious T cells prevail, whereas in MIC_Cop_-treated animals Tregs control the scenario. Apart, from CD4^+^CD25^+^FoxP3^+^ other regulatory cell subsets may play their roles and should be envisaged in future studies.

## Conclusions

Donor MIC cell therapy is currently being implemented in living donor kidney transplantation [21]. The findings of the present study, although not clarifying all mechanistic questions, constitute a sound basis for a clinical phase I study with MIC_Cop_ cells in patients with MS.

## Abbreviations

Cop: copaxone^®^
CFA: complete Freund’s adjuvant
CNS: central nervous system
DCs: dendritic cells
EAE: experimental autoimmune encephalomyelitis
GA: glatiramer acetate
IFA: incomplete Freund’s adjuvant
LNCs: lymph node cells
MBP: myelin basic protein
MMC: mitomycin C
MICs: mitomycin C-induced cells
MIC_Cop_: copaxone^®^-loaded MMC-induced cells
MOG: myelin oligodendrocyte glycoprotein
MS: multiple sclerosis
MSCs: mesenchymal stromal cells
MST: mean survival time
OVA: ovalbumin
PBMCs: peripheral blood mononuclear cells
PHA: phytohemagglutinin
PLP: proteolipid protein
SD: standard deviation
SEM: standard error of the mean
SPCs: splenocytes
tolDCs: tolerogenic DCs
UV/C: ultraviolett C
